# Replacing Barley and Soybean Meal With By-products, in a Pasture Based Diet, Alters Daily Methane Output and the Rumen Microbial Community *in vitro* Using the Rumen Simulation Technique (RUSITEC)

**DOI:** 10.3389/fmicb.2020.01614

**Published:** 2020-07-22

**Authors:** Paul E. Smith, Sinéad M. Waters, David A. Kenny, Tommy M. Boland, John Heffernan, Alan K. Kelly

**Affiliations:** ^1^UCD School of Agricultural and Food Science, University College Dublin, Dublin, Ireland; ^2^Teagasc Animal and Bioscience Research Department, Teagasc Grange, Meath, Ireland

**Keywords:** rumen microbiome, by-products, RUSITEC, methane, 16S rRNA

## Abstract

Plant based by-products (BP) produced from food and bioethanol industries are human inedible, but can be recycled into the global food chain by ruminant livestock. However, limited data is available on the methanogenesis potential associated with supplementing a solely BP formulated concentrate to a pastoral based diet. Therefore the objective of this *in vitro* study was to investigate the effects of BP inclusion rate (in a formulated concentrate) to a pasture based diet on dietary digestibility, rumen fermentation patterns, methane production and the prokaryotic microbial community composition. Diets consisted of perennial ryegrass and one of two supplementary concentrates, formulated to be isonitrogenous (16% CP) and isoenergetic (12.0 MJ/ME/kg), containing either 35% BP, barley and soybean meal (BP35) or 95% BP (BP95) offered on a 50:50 basis, however, starch, NDF and fat content varied. The BPs, included in equal proportions on a DM basis, were soyhulls, palm kernel expeller and maize dried distillers grains. The BP35 diet had greater (*P* < 0.05) digestibility of the chemical constituents DM, OM, CP, NDF, ADF. Greater total VFA production was seen in the BP35 diet (*P* < 0.05). Daily methane production (mmol/day; +22.7%) and methane output per unit of total organic matter digested (MPOMD; +20.8%) were greatest in the BP35 diet (*P* < 0.01). Dietary treatment influenced microbial composition (PERMANOVA; *P* = 0.023) with a greater relative abundance of *Firmicutes* (adj *P* < 0.01) observed in the BP35. The Firmicutes:Bacteroidetes ratio was significantly reduced in the BP95 diet (*P* < 0.01). The relative proportions of *Proteobacteria* (adj *P* < 0.01), *Succinivibrionaceae* (adj *P* < 0.03) and *Succinivibrio* (adj *P* = 0.053) increased in the BP95 diet. The abundance of *Proteobacteria* was found to be negatively associated with daily methane production (r_s_, −0.71; *P* < 0.01) and MPOMD (r_s_, −0.65; *P* < 0.01). Within *Proteobacteria*, the relationship of methane production was maintained with the mean abundance of *Succinivibrio* (r_s_, −0.69; *P* < 0.01). The abundance of the *Firmicutes* phyla was found to be positively correlated with both daily methane production (r_s_, 0.79; *P* < 0.001) and MPOMD (r_s_, 0.75; *P* < 0.01). Based on *in vitro* rumen simulation data, supplementation of an exclusively BP formulated concentrate was shown to reduce daily methane output by promoting a favorable alteration to the rumen prokaryotic community.

## Introduction

The processing of crops for the global food, oil and ethanol industries generates a source of residual by-product (BP) plant matter which can be utilized as animal feed (Mirzaei-Aghsaghali and Maheri-Sis, 2008). Such BPs contain little economic value as edible food for human consumption due to safety, quality and digestibility considerations (Grasser et al., 1995). However, BPs contain digestible nutrients that can be used as feed by the livestock industry. In particular, ruminant animals, through microbial fermentation, have the ability to digest cellulose and utilize highly fibrous feed sources (Sasson et al., 2017) which is often typical of such BPs. As a result, ruminants have the unique ability to convert these human inedible low value raw ingredients, into high quality animal dairy and meat proteins for human consumption.

Grazed pasture is usually the most economical feed source for ruminants in temperate regions such as Ireland (Finneran et al., 2012) although strategic concentrate usage is common throughout the ruminant production cycle (O’Brien et al., 2018) in order to overcome deficiencies in nutrient supply. Concentrates are utilized to supplement the diet of dairy cows during bad weather conditions and poor grass growth in order to ensure adequate energy intake to support milk production and quality (McGrath, 2013). Feeding concentrates is also a key component of beef production systems, especially during the winter finishing period (McGee, 2005). Currently, near 10% of the global area dedicated to cereal production, is used to produce grain for ruminant diets (Mottet et al., 2017). The inefficiency with which human edible feed is converted into ruminant derived protein and energy (Wilkinson, 2011) coupled with the need to feed a growing global population, has therefore brought into question the strategy of supplementing ruminant diets with human edible sources of feed.

Replacing human edible cereals with BP feeds, offers a more sustainable and efficient use of supplemented feed in pastoral based ruminant production systems (Wilkinson, 2011). However, the nutritional value between individual BPs can vary (Lee et al., 2003; Kim et al., 2013) and therefore must be accounted for when considering their inclusion in dietary formulations. Nonetheless, members of our group have shown the formulation of concentrates, formulated solely from dried distillers grains and solubles (DDGS), palm kernel expeller (PKE) and soyhulls (SH) supplemented to a pasture based dairy system, to produce a similar level of milk output, to grain formulated concentrates (Whelan et al., 2016; Condren et al., 2019). However, data on the effects of this BP concentrate on the rumen microbiome and methane production is needed to further assess the sustainability of the formulation.

Both the microbial population residing in the rumen (Morgavi et al., 2010; Henderson et al., 2015; Tapio et al., 2017) and enteric methane production (Beauchemin et al., 2008, 2020; Martin et al., 2010) has been shown to be altered by the composition of the diet offered to ruminants. Indeed, certain BP feeds are known to be lower in starch and higher in fiber than conventional cereal and oilseed grains (Ertl et al., 2015) leading to shifts in ruminal fermentation patterns and digestion end products. Thus, this highlights the importance of investigating, in tandem, the effects of replacing grain based concentrates with formulations derived from BPs, on the rumen microbiome and methane production.

On an individual BP bases, the high lipid content of DDGS have been shown to reduce methane output in beef and dairy cattle, in comparison to cereal and soybean meal based diets (McGinn et al., 2009; Benchaar et al., 2013; Hünerberg et al., 2013). Indeed, the lipid proportion of DDGS has been shown to decrease the abundance of members of the fibrolytic microbial community in some (Ramirez et al., 2012; Castillo-Lopez et al., 2014) but not all studies (Castillo-Lopez et al., 2017). Additionally, PKE has shown methane abatement potential through reductions to the methanogen population *in vivo* (Abubakr et al., 2014) and production of methane *in vitro* (Lee et al., 2003; Kim et al., 2013).

Therefore, this experiment was designed to evaluate, the effects of supplementing a solely BP formulated concentrate, with increased dietary fiber and fat, on rumen fermentation parameters, methane production and the microbial community composition with perennial ryegrass pasture as the main forage source *in vitro*.

## Materials and Methods

### Experimental Licensing and Apparatus

All procedures described in this experiment were approved by the animal research ethics committee (AREC) at University College Dublin (UCD) and conducted under the European directive 2010/63/EU and S.I. No. 543 of 2012. Each person who carried out procedures on experimental animals, during the course of this experiment, was licensed to do so by means of individual authorisation from the Health Product Regulatory Authority (HPRA). Two rumen simulation technique (RUSITEC) systems with eight vessels/system (Sanshin Industrial Co. Ltd., Yokohama, Japan), were used to simulate the rumen environment. Each fermenter had a nominal volume of 850 ml with the general incubation procedure described by Czerkawski and Breckenridge (1977).

### Experimental Design, Rumen Inoculum and Diets

Dietary treatments consisted of a perennial ryegrass *(Lolium perenne)* based pasture (PRG) and one of two supplementary concentrates containing either barley, soybean meal and 35% BP (BP35) or solely 95% BP (BP95) offered on a 50:50 basis (Table 1). The BPs, included in equal proportions on a dry matter (DM) basis, were SHs, PKE and maize DDGS. The PRG component of each treatment was harvested in July 2015 from the grazing platform at UCD Lyons Estate Farm, Celbridge, Kildare, Ireland (53°17 ′56 ′ ′N, 6°32 ′18 ′ ′W). Samples were cut to a height of 4 cm from the same pasture using a handheld shears (Gardena Accu 90; Gardena GmbH, Ulm, DE) and subsequently stored at −20°C. Concentrates were formulated to be isonitrogenous [16% crude protein (CP)] and isoenergetic (12.0 MJ/ME/kg), however, starch, NDF and fat content varied. The BP35 concentrate was targeted to contain 280 g of starch and 270 g of neutral detergent fiber (NDF) per kg of DM with the BP95 containing 30 g of starch and 500 g of NDF per kg of DM, respectively. A crude fat content, based on ether extract (EE), was targeted at 2.51 and 5.6% for the BP35 and BP95 diets, respectively.

**TABLE 1:** Chemical composition of concentrates and grass along with ingredient inclusion rate of concentrates fed during the experiment.

	BP35	BP95	Pasture
**Chemical composition (% of DM unless stated)**			
Dry matter	90.5	92.1	21.6
Crude protein	16.85	16.73	25.01
Neutral detergent fiber	32.22	53.37	46.79
Acid detergent fiber	15.89	31.76	21.31
Water soluble carbohydrates	0	0	15.5
Starch (g)	200	30	0
Ether extract	2.51	5.6	3.1
Ash	7.35	7.75	10.26
Gross energy (MJ/kg DM)	16.92	17.73	16.97
**Ingredient inclusion rate of concentrates**			
Barley	45.0	0.0	–
Soybean meal	12.0	0.0	–
Distillers dried grain	11.6	31.0	–
PKE	11.6	31.0	–
Soybean hulls	11.6	31.0	–
Molasses	5.0	5.0	–
Calcined magnesite	0.8	0.8	–
Salt	0.7	0.7	–
Palm oil	0.6	0.6	–
Lime flour	0.5	0.2	–
Mono-calcium di-phosphate	0.3	0.0	–
Vitamin and mineral premix	0.5	0.5	–

*BP35 = Ration containing 35% by-products. BP95 = Ration containing 95% by-products. Vitamin and mineral premix contained: 33.9% Ca, 500 mg Co/kg, 7,400 mg Cu/kg, 2,000 mg I/kg, 130 mg Se/kg, 10,000 mg Mg/kg, 25,000 mg Zn kg, 1600,000 iu Vitamin A/kg, 400,000 iu Vitamin D3/kg and 2,000 mg Vitamin E/kg.*

Dietary treatments were randomly allocated to sixteen fermentation vessels and added to each vessel in nylon bags (100-μm pore size). Samples of PRG were removed from storage (−20°C) and immediately chopped to 1–2 cm in length with a bowl chopper before being added to each nylon bag and subsequently being refrozen and stored (−20°C). For each treatment, 10 g DM of either concentrate under investigation and the fresh weight equivalent of 10 g DM of PRG were added to separate nylon bags.

Rumen inoculum was obtained from three ruminally cannulated beef heifers of continental breed crosses, offered grass silage (18% DM, 13.37% CP, 51.5% NDF and 37.39% acid detergent fiber (ADF)) and concentrate (86% DM, 16% CP) in a 50:50 ratio, in order to support estimated maintenance energy requirements (Agriculture Food Research Council, 1993). Rumen fluid collection took place before morning feeding at 09:00.

### Experimental Procedure

A single *in vitro* incubation period of 14 days, using the first 10 days for microbial adaptation and fermentation stabilization (Jaurena et al., 2005) and the last 4 for sampling was implemented using the RUSITEC. On Day 1 of the experiment, solid digesta and rumen fluid were collected from the three ruminally cannulated heifers, with the fluid strained through four layers of cheesecloth. The samples were pooled, flushed with carbon dioxide (CO_2_) and incubated at 39°C before being transferred to the RUSITEC vessels within 30 min of collection. Each vessel was inoculated with 500 ml of rumen fluid and 350 ml of artificial saliva (McDougall, 1948). Nylon bags containing PRG were removed from storage (−20°C), on the day prior to incubation, and allowed to thaw for 24 h. Treatments were added to each vessel in individual nylon bags (*n* = 2) containing PRG and the BP concentrate under investigation. In addition, a third nylon bag, containing 70 g of rumen solid digesta, was added to each vessel. This resulted in each vessel containing a total of three nylon bags (PRG, concentrate and rumen digesta) at the beginning of the experiment. Artificial saliva was prepared daily and was continuously infused at a rate of 640 ml/d (dilution rate of 3.33%/h) using the Watson-Marlow 500 series peristaltic pump (Watson-Marlow Fluid Technology Group, Cornwall, United Kingdom). The displaced effluent and fermentation gasses from each vessel were collected into effluent bottles and gas collection bags, respectively. On day 2, each vessel was opened and nylon bags containing rumen digesta solids and the concentrates under investigation were removed, squeezed and washed in artificial saliva. The liquid fractions of the washings were returned to each vessel along with two new nylon bags, containing PRG and the concentrate under examination. Throughout the 14-day period, nylon bags containing PRG were incubated for a 48 h period after which they were replaced by a new nylon bag containing forage. Nylon bags containing either concentrate under investigation were replaced every 24 h by new bags containing the same concentrate. During the adaptation period effluent outflow and gas production were measured, as well as pH of the fermentation vessels.

### Diet Digestibility, Rumen Fermentation and Methane Production

Dry matter degradation, gas production and outflow of fermentation products were measured on days 11, 12, 13, and 14. Overflow vessels were kept in cold water (2°C) to stabilize fermentation products. A 4 ml sample of outflow liquor, from each vessel, was preserved in 1 ml of 50% trichloroacetic acid (TCA), and stored at −20°C for volatile fatty acid (VFA) and ammonia (NH_3_-N) analysis.

After removal from fermenters, nylon bags were rinsed with cold water. Feed residues in the nylon bags were washed in a domestic washing machine using the cold rinse cycle in the absence of detergent (30 min) to remove the loosely attached bacteria. The feed residues were dried in a 55°C forced air oven and weighed. Feed dry matter digestibility (DMD) was calculated as the amount of material that had disappeared from the nylon bags after 24 and 48 h of incubation, for concentrates and forage respectively. Chemical composition of the dried incubation residues was determined to calculate digestibility of feed components.

Gas was measured using gas reusable polyethylene bags fitted with one way valves. These were placed on each outflow vessel to measure gas production including methane percentage. Gas volume was quantified by manual expulsion through the dual diaphragm DC-1 dry gas test meter (Sinagawa Corp. Tokyo, Japan) with the percentage of methane estimated using the infra-red ADC SB2000 wall mounted analyser (ADC Gas Analysis Ltd., Hoddeston, United Kingdom) (O’Connor et al., 2019a, b). Daily calibrations of the ADC SB2000 were conducted with a 10% methane span gas.

### Chemical Analysis and VFA Production

The DM content (g/kg) of the feed and feed residue samples were determined after drying the samples at 55°C for 72 h in a forced air oven. Dried feed and feed residue samples were then ground in a hammer mill fitted with a 1-mm screen for subsequent chemical analysis (Lab Mill, Christy Turner, Suffolk, United Kingdom). Ash concentrations (g/kg DM) were determined by complete combustion in a muffle furnace (Nabertherm, GmbH, Lilienthal, Germany) at 550°C for 4 h. The nitrogen concentration (g/kg DM) of feed was determined using a LECO FP 528 instrument (Leco Instruments UK Ltd., Stockport, United Kingdom). The nitrogen concentration of the feed was multiplied by 6.25, to determine CP concentrations (g/kg DM). Determination of NDF and ADF concentrations were determined by the method of Van Soest et al. (1991) using the ANKOM220 Fiber Analyzer (ANKOM Technology, Macedon, NY, United States). Grass and concentrate samples were analyzed for NDF with sodium sulphite and with a heat stable amylase included for concentrate samples only. Both NDF and ADF are expressed inclusive of residual ash (g/kg DM). Gross energy was determined on pelletized samples using a bomb calorimeter (Parr Instrument Company, Moline, IL, United States). The EE content was determined using Soxtec instruments (Tecator, Höganäs, SE) and light petroleum ether. The chemical composition of the concentrates and PRG are presented in Table 1. Samples of vessel effluent were thawed for 16 h to 4°C before being centrifuged for 10 min (1600 *g*; 4°C). A 1 ml sample of supernatant was drawn off and diluted one in five with distilled water (dH_2_O) and centrifuged for 15 min (1600 *g*; 4°C). Following this 200 and 250 μl of supernatant was drawn off into separate test tubes for NH_3_-N and VFA analysis, respectively. Concentrations of NH_3_-N were determined using the phenol-hypochlorite method of Weatherburn (1967) using a Shimadzu UV/Vis UVmini-1240 spectrophotometer (Shimadzu UK Ltd., Wolverton Mill South, Milton Keynes, United Kingdom). For VFA analysis, the 250 μl of supernatant was diluted with 3.75 ml of dH_2_O and 1 ml of internal standard (0.5 g 3-metyl-*n*-valeric acid in one liter of 0.15 M oxalic acid). Following this, diluted VFA samples were centrifuged for 5 min (260 *g*; 21°C) followed by filtration through a 0.45 μm filter (Cronus Syringe filter PTFE 13 mm; SMI-LabHut Ltd., Maisemore, Gloucester, United Kingdom) into a 4 ml GC vial (Thermo Scientific, Langerwehe, Germany) and stored (−20°C) until analysis was conducted. VFAs were quantified by injecting 1 μl, via an auto sampler, onto a 25 m × 0.53 mm i.d. megabore Varian gas chromatograph (GC) 3800 column, [coating CP-Wax 58 (FFAP) – CB (no. CP7614)] (Varian, Middelburg, Netherlands). An initial injector temperature of 75°C, immediately rose to 95°C, followed by a rate of temperature increase of 3°C/min up to 200°C (held for 50 s). Nitrogen was used as a carrier gas. The pressure of the column was held at 2.3 psi and the column rate was 8.1 ml/min. Estimates of hydrogen (H) recovery within each vessel were calculated based on the concentration of individual VFAs as described by Marty and Demeyer (1973) with the exclusion of hydrogen gas (H_2_).

### Microbial DNA Extraction

On the final day of incubation, prior to opening each vessel, a 15 ml sample of fluid was collected via an aspiration port at the top of each RUSITEC vessel. Fluid samples were collected in individual disposable sterile syringes and deposited in 15 ml falcon tubes. Immediately after collection, samples of vessel effluent were snap frozen in liquid nitrogen and subsequently stored at −80°C. Under liquid nitrogen, samples were ground to a fine powder using a pestle and mortar. Microbial DNA was extracted from approximately 250 mg of ground sample using the repeated bead beating and column purification method (Yu and Morrison, 2004). DNA quality was assessed on 0.8% agarose gels with the concentration of extracted DNA quantified on the Nanodrop 1000 spectrophotometer.

Microbial DNA extractions were also performed on the ZymoBIOMICS^TM^ Microbial Community Standard (MC) (Zymo Research Corp., Irvine, CA, United States) to assess DNA extraction performance. Extractions were performed on the ZymoBIOMICS^TM^ MC in triplicate and were treated as an internal control and subject to the same library preparation and sequencing regime as samples collected from the RUSITEC systems.

### Library Preparation and Sequencing

Using 12.5 ng of individual microbial DNA obtained from the RUSITEC vessels, 16 amplicon libraries were generated by performing two rounds of PCR amplification as outlined in the Illumina Miseq 16S Sample Preparation Guide with minor modifications to cycle length, as outlined by McGovern et al. (2018a). An additional four amplicon libraries were generated to assess sequencing run performance and library preparation. Three amplicon libraries were generated from the extractions performed on the ZymoBIOMICS^TM^ MC with a final library generated using the ZymoBIOMICS^TM^ Microbial Community DNA Standard (DS) (Zymo Research Corp., Irvine, CA, United States) to assess library preparation and sequencing run performance. This resulted in a total of 20 amplicon libraries being generated (RUSITEC *n* = 16; MC *n* = 3; DS *n* = 1).

The first round of PCR amplification, targeting the V4 hypervariable region of the 16S rRNA gene, was performed using the 515F/806R primers (Caporaso et al., 2011), designed with Nextera over hang adapters, and 2x KAPA Hifi HotStart ReadyMix DNA polymerase (Roche Diagnositics, West Sussex, United Kingdom). Cycle conditions were as follows: 95°C for 3 min, 20 cycles at 95°C for 30 s, 55°C for 30 s, 72°C for 30 s and then 72°C for 5 min.

Amplicons were purified using the MinElute PCR Purification Kit (Qiagen, Manchester, United Kingdom). Following purification, amplicons were subject to a second round of PCR to permit attachment of dual indices and Illumina sequencing adapters using the Nextera XT indexing kit (Illumina, San Diego, CA, United States). Cycle conditions for the second round of PCR were 95°C for 3 min, 8 cycles at 95°C for 30 s, 55°C for 30 s, 72°C for 30 s and then 72°C for 5 min followed by an additional PCR purification with the MinElute PCR Purification Kit (Qiagen, Manchester, United Kingdom). Confirmation of amplicon generation was conducted visually on a 2% agarose gel. Amplicons were pooled together in equal concentration and subject to gel purification using the QIAquick Gel Extraction Kit (Qiagen, Manchester, United Kingdom) to remove adapter primers and further purified to remove any residues of agarose using the MinElute PCR purification kit (Qiagen, Manchester, United Kingdom).

Pooled sample purity and quantity was analyzed on the Nanodrop 1000 with further validation on the Qubit fluorometer and using the KAPA SYBR FAST qPCR universal kit with Illumina Primer Premix (Roche Diagnositics, West Sussex, United Kingdom). Following this, the library pool was diluted and denatured as per the Illumina Miseq 16S Sample Preparation Guide with sequencing conducted on the Illumina MiSeq using the 500 cycle version 2 MiSeq reagent kit (Illumina, San Diego, CA, United States).

### Sequencing Analysis

Amplicon sequence data was processed in *R* (version 3.5.2) using *DADA2* (version 1.11.3) and submitted to the pipeline as previously described (Callahan et al., 2016) with minor modifications. Read quality was determined based on the visualization of Q scores with the aim to ensure mean Q scores of >30 were upheld for forward and reverse reads. To achieve this, forward reads were trimmed to a length of 240 bp and reverse reads trimmed to 200 bp. The removal of primer sequences was conducted using the trimLeft function. Identical sequences were combined using the dereplication function followed by the merging of forward and reverse reads. Following this an amplicon sequence variant (ASV) table was constructed after which chimeric sequences were removed. The assignTaxonomy function was used to assign taxonomy to each ASV using the RefSeq + RDP database (NCBI RefSeq 16S rRNA database supplemented by RDP) downloaded from the *DADA2* website^1^. A bootstrapping threshold of 80 was applied with the addition of the minBoot = 80 function. Sample metadata, sequence taxonomy and ASVs were combined into a phyloseq object using *phyloseq* (version 1.24.2) (McMurdie and Holmes, 2013) for further analysis. Predictions of metabolic pathways for each sample, based on the generated ASVs, were conducted using *CowPI* (Wilkinson et al., 2018).

### Statistical Analysis

Data were checked for normality and homogeneity of variance by histograms, qqplots, and formal statistical tests as part of the UNIVARIATE procedure of SAS (version 9.1.3; SAS Institute). Data that were not normally distributed were transformed by raising the variable to the power of lambda. The appropriate lambda value was obtained by conducting a Box-Cox transformation analysis using the TRANSREG procedure of SAS. *In-vitro* data were analyzed using a mixed model ANOVA (PROC MIXED). Fixed effects in the model included BP concentrate (BP35 or BP95). Fermentation vessel was included as a random effect. Differences between treatments were determined by F-tests using Type III sums of squares. The PDIFF command incorporating the Tukey test was applied to evaluate pairwise comparisons between treatment means.

The generated ASV table and sequence taxonomies were analyzed in *R* (version 3.4.2). Of the 16 samples sequenced from the dietary treatments, one sample from the BP35 group was excluded from the analysis due to having a substantially lower sequencing depth compared to all other samples leaving a total of 15 samples for analysis (BP35 *n* = 7; BP95 *n* = 8). The relative abundance of taxa was calculated for each sample at the genus level in phyloseq. A PERMANOVA test, based on 9,999 permutations and a significance level of *P* < 0.05, was carried using the *R* package vegan (Oksanen et al., 2019) (version 2.5.4) implemented through *microbiome* (Leo et al., 2017) (version 1.0.2) to investigate differences in the community structure amongst samples based on treatment at the level of genus. The Wilcoxon rank sum test, with Benjamini Hochberg (BH) correction for false discovery rate (FDR) was implemented for identification of significant treatment differences in relative abundance of taxa based on adjusted *P* values (adj *P*) of <0.05. The ZymoBIOMICS^TM^ MC standard contains eight bacteria and two yeast. As a result the mean relative abundance of the eight most abundant ASVs, within the three MC controls, was used to analyze performance of the DNA extraction method. Spearman’s rank correlation coefficient was used to determine consistency of the internal positive controls and correlations between microbial abundances and production data. A Student’s *t*-test was used to compare diversity metrics for microbial community structure comparisons and the ratio of Firmicutes:Bacteroidetes. Statistical analysis of predicted pathways was carried out using STAMP (version 2.1.3; Parks et al., 2014). Comparison of predicted pathways was conducted using the proportion of reads that were annotated to each individual metabolic pathway as a percentage of the total reads (read relative abundance). Statistical analysis of read relative abundance was conducted using a Student’s *t*-test, with BH correction for FDR (adj *P* < 0.05).

## Results

### Diet Digestibility and Fermentation Parameters

The disappearance of DM and its chemical constituents are given in Table 2. The BP35 diet had greater (*P* < 0.05) digestibility of the chemical constituents DM, organic matter (OM), CP, NDF, ADF compared to the BP95 diet. The fermentation parameters including VFA production per day and molar proportions for each dietary treatment are presented in Table 3. No significant difference was recorded in pH between treatments (*P* = 0.132). The BP35 diet had greater total VFA production than BP95 (*P* < 0.05). Diet did not alter (*P* > 0.10) the VFA proportions of either acetic or propionic acid, nor acetate:proprionate ratio. The proportion of butyric acid was greatest in the BP35 diet compared to the BP95 (*P* < 0.01). There was no effect of diet type on NH_3_-N output per day (*P* = 0.429).

**TABLE 2:** Mean effects of by-product inclusion rate on disappearance of feed and its components in the rumen simulation technique (RUSITEC) system.

Parameter	BP35	BP95	SEM	*P* value
Dry matter (c)	69.67^a^	57.28^b^	0.62	<0.0001
Dry matter (t)	78.44^a^	67.50^b^	0.89	<0.0001
Organic matter (c)	65.36^a^	52.75^b^	0.51	<0.0001
Organic matter (t)	75.75^a^	66.75^b^	0.61	<0.0001
Crude protein (c)	75.32^a^	63.96^b^	0.84	<0.0001
Crude protein (t)	86.33^a^	80.62^b^	0.44	<0.0001
NDF (c)	37.21^a^	45.86^b^	0.75	<0.0001
NDF (t)	63.10^a^	58.46^b^	0.89	<0.0001
ADF (c)	24.23^a^	34.41^b^	0.92	<0.0001
ADF (t)	54.27^a^	50.06^b^	1.15	0.0096

*Digestibility: c = Concentrate component of overall diet (i.e., 50% of overall DM content of diet); t = Total diet (i.e., 50% concentrate component and 50% forage component on a DM basis). BP35 = Ration containing 35% by-products. BP95 = Ration containing 95% by-products. SEM = Standard error of the mean. ^*a*,*b*^Means within a row with different superscript differ (P < 0.05).*

**TABLE 3:** Mean effects of by-product inclusion rate on pH and fermentation pattern in the rumen simulation technique (RUSITEC) system.

Parameter	BP35	BP95	SEM	*P* value
Daily gas production (l/d)	1.97^*a*^	1.68^*b*^	0.07	0.0388
Daily methane production (mmol/day)	3.92^*a*^	3.03^*b*^	0.10	<0.0001
Methane production (mmol/omd)	0.30^*a*^	0.24^*b*^	0.01	<0.0001
**Fermentation parameters**				
pH	6.72	6.79	0.04	0.1323
NH_3_-N (mg/d)	144.51	140.46	3.52	0.4291
**VFA production (mmol/day)**				
Total volatile fatty acids	38.36^*a*^	35.73^*b*^	1.26	0.0137
**VFA molar proportion (%)**				
Acetic: propionate ratio	3.55	3.57	0.073	0.8711
Acetic	63.94	64.65	0.003	0.2075
Propionic	18.02	18.14	0.003	0.7882
Butryic	14.94^*a*^	13.40^*b*^	0.005	0.0039
**Estimated H parameters**				
H production (mmol/day)*	75.91	70.01	2.41	0.0604
H incorporated (mmol/day)*	41.82^*a*^	36.46^*b*^	0.97	0.0015
H recovery (%)*	55.15^*a*^	52.22^*b*^	0.93	0.0431

BP35 = Ration containing 35% by-products. BP95 = Ration containing 95% by-products. SEM = Standard error of the mean. ^*a,b*^Means within a row with different superscript differ (P < 0.05). *H production, incorporation and recovery was calculated from the concentration of individual VFAs as per Marty and Demeyer (1973) without the inclusion of H_2_.

### Daily Gas and Methane Production

**FIGURE 1:**
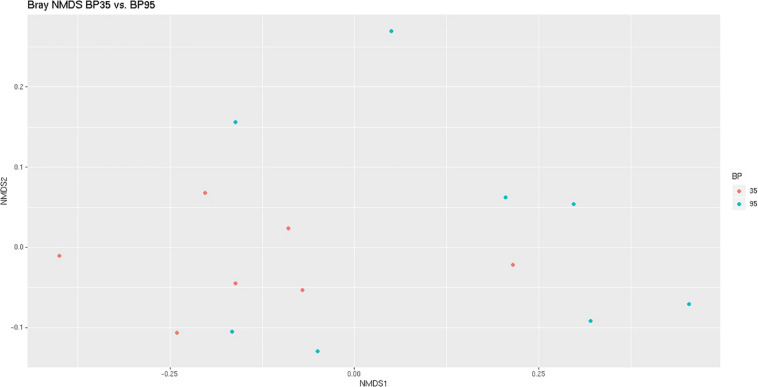
Bray Curtis NMDS plot highlighting differences in the community composition between diets. Different color dots represent samples obtained from rumen simulation technique (RUSITEC) systems incubated with different diets. Red = Concentrate formulated with 35% by-product (BP35), Blue = Concentrate formulated with 95% by-product (BP95).

**FIGURE 2:**
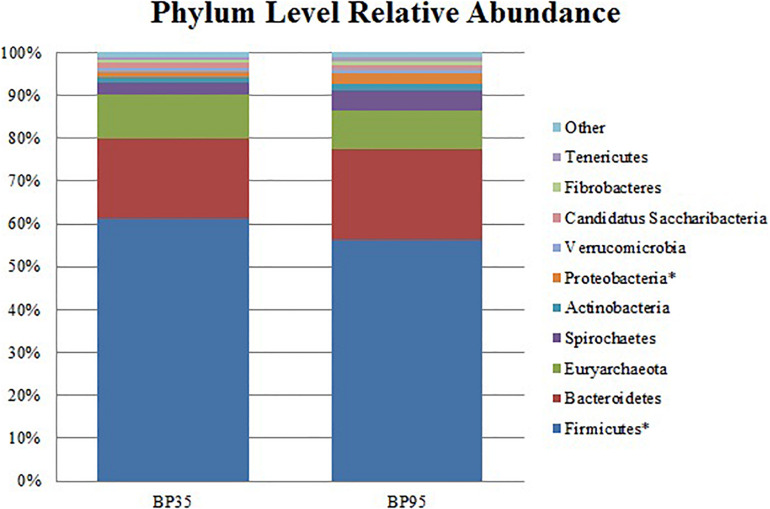
Stack plot comparing the differences, between diets, in the relative abundances of the ten most abundant microbes at the phylum level. Asterisk (^∗^) signifies phyla with a significantly different relative abundance between diets. BP35 = Concentrate formulated with 35% by-product. BP95 = Concentrate formulated with 95% by-product.

Average daily gas and methane production was altered between the treatment groups (*P* < 0.05). A greater mean daily methane output (mmol/day) was observed in the BP35 compared to the BP95 diet (*P* < 0.01) (Figure 1). Increased methane output with the BP35 diet persisted when expressed per unit of total organic matter digested (OMD; *P* < 0.001) (Figure 2). Daily gas production (l/day) was greatest in the BP35 diet (*P* < 0.05). Based on VFA analysis, there was a tendency for an increase in the production of theoretical H in the BP35 diet (*P* = 0.06). A reduction in the H incorporation (*P* < 0.01) and recovery (*P* < 0.05) was observed in the BP95 group.

### Sequencing Performance

After quality filtering, merging and removal of chimeric sequences, a total of 1,411,464 reads were generated with a mean of 70,573.2 ± 16,920.78 reads per sample. The eight most abundant ASVs in the MC control accounted for 97.78 ± 0.24% of sequences. The mean correlation coefficient between the composition of libraries generated from the MC standards, and the theoretical composition of the ZymoBIOMICS^TM^ standard was (r_s_, 0.79; *P* < 0.03). In addition, a strong positive correlation between the DS standard and the theoretical composition of the ZymoBIOMICS^TM^ standard was observed (r_s_, 0.95; *P* < 0.001). Performance of the extraction method, library preparation and the sequencing run were deemed adequate based on correlations of our internal controls, and that of theoretical composition of the ZymoBIOMICS^TM^ standards above that previously reported by members of our group (McGovern et al., 2018b).

### Microbial Community Structure and Composition

Across all samples, the relative abundance of bacteria (91.05%) was greater in comparison to archaea (8.95%). At the phylum level, *Firmicutes* dominated preceded by *Bacteroidetes* and *Euryarchaeota*. At the genus level, 171 different genera of bacteria and archaea were identified, however, only those with a relative abundance greater than 0.01% are reported. *Methanobrevibacter* (11.42%) was the most abundant microbe followed by *Prevotella* (10.32%). Other highly abundant bacteria included *Lactobacillus*, *Streptococcus*, *Treponema* and *Butyrivibrio*. After *Methanobrevibacter*, other highly abundant methanogens included *Methanosphaera*, *Methanobacterium*, *Methanomassiliicoccus*, *Methanimicrococcus* and *Methanomicrobium*.

### Effect of By-product Inclusion Rate on Microbial Community

A NMDS plot of sample data generated using Bray Curtis dissimilarity analysis (Figure 1) displayed a moderate degree of clustering of samples based on diet, particularly BP35, with respect to the microbial communities. Based on the results of a PERMANOVA test, BP inclusion rate was deemed to have altered the microbial composition between dietary treatments (*P* = 0.023).

Differences in the alpha diversity of the microbial community structure, between the groups, was not observed when comparisons were conducted using the Shannon Index (*P* = 0.76). However, there was a tendency for a decrease in microbial diversity in the BP95 when diversity investigations were conducted with the Simpson metric (0.989 vs. 0.991; *P* = 0.08).

**FIGURE 3:**
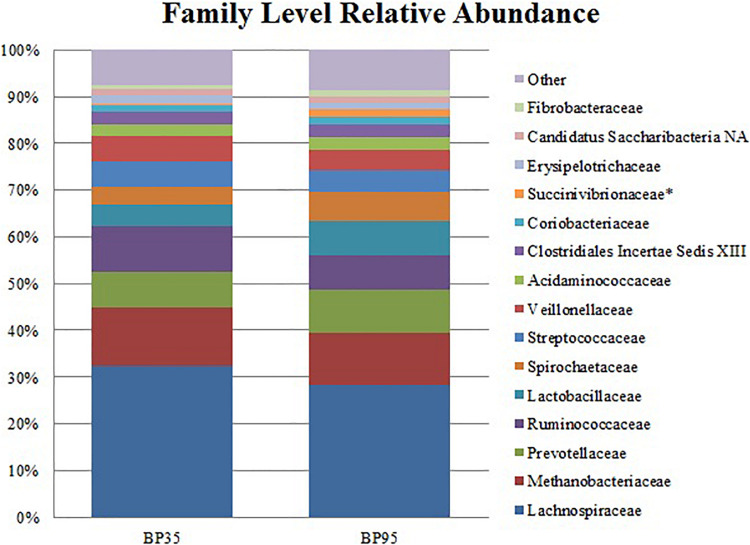
Stack plot comparing the differences, between diets, in the relative abundances of the fifteen most abundant microbes at the family level. Asterisk (^∗^) signifies families with a significantly different relative abundance between diets. BP35 = Concentrate formulated with 35% by-product. BP95 = Concentrate formulated with 95% by-product.

Comparisons of the ten and fifteen most abundant microbes classified at the phylum and family level are displayed in Figures 2, 3, respectively. A dietary effect was observed at the phylum level (Table 4), with a greater relative abundance of *Firmicutes* (adj *P* < 0.01) observed in the BP35 with an increased proportion of *Proteobacteria* in BP95 diet (adj *P* < 0.01). In addition, there was a tendency for an increased relative abundance of the *Bacteroidetes* phylum in the BP95 diet (adj *P* = 0.084). The increased proportion of bacteria, belonging to the phyla *Proteobacteria*, persisted at lower taxonomic ranks. For example, an increased abundance of *Succinivibrionaceae* (adj *P* < 0.03) at the family level with a strong tendency toward a statistically significant rise in the relative abundance of *Succinivibrio* (adj *P* = 0.053) at the genus level (Table 5), was observed in the BP95 diet. The Firmicutes:Bacteroidetes ratio was reduced in the BP95 diet (*P* < 0.01).

**TABLE 4:** Mean relative abundance (%) of bacteria significantly different between diets.

	BP35 (%)	BP95 (%)	*P* value	adj *P* value
**Phylum**				
*Firmicutes*	61.40^a^	56.42^b^	<0.001	<0.01
*Proteobacteria*	1.20^a^	2.44^b^	<0.001	<0.01
**Family**				
*Succinivibrionaceae*	0.42^a^	1.54^b^	<0.001	0.03

*BP35 = Concentrate formulated with 35% by-product. BP95 = Concentrate formulated with 95% by-product. *P* values were generated using the Wilcoxon rank sum test, with correction for FDR using the BH method. Adjusted (adj) *P* values deemed significant when (*P* < 0.05).*

**TABLE 5:** Mean relative abundance of the 33 most abundant genera accounting for >90% of the relative abundance across both diets.

Genus	BP35 (%)	BP95 (%)	*P* value	*adj P* value
*Alistipes*	0.90	0.89	0.955	0.996
*Anaeroplasma*	0.87	0.99	0.232	0.629
*Anaerovibrio*	3.04	2.06	0.014	0.251
*Anaerovorax*	1.79	1.87	0.694	0.899
*Bacillus*	0.64	0.62	0.694	0.899
*Bacteroides*	1.83	3.20	0.029	0.291
*Butyrivibrio*	4.30	4.37	0.536	0.797
*Clostridium IV*	0.76	0.25	0.001	0.053
*Clostridium XlVa*	3.10	3.02	0.955	0.996
*Eubacterium*	1.58	1.85	0.694	0.899
*Fibrobacter*	1.03	1.47	1.000	1.000
*Intestinimonas*	1.33	1.20	0.397	0.709
*Lachnospiracea incertae sedis*	2.63	2.66	0.779	0.972
*Lactobacillus*	6.04	8.46	0.094	0.479
*Methanobacterium*	1.10	0.84	0.094	0.479
*Methanobrevibacter*	12.91	10.12	0.094	0.479
*Methanosphaera*	2.14	2.25	0.536	0.797
*Mogibacterium*	1.89	1.53	0.094	0.479
*Olsenella*	0.50	0.69	0.281	0.679
*Prevotella*	9.76	10.81	0.189	0.620
*Pseudobutyrivibrio*	2.39	2.17	0.536	0.797
*Ruminococcus*	3.10	2.08	0.021	0.251
*Saccharibacteria genera incertae sedis*	1.80	1.73	1.000	1.000
*Schwartzia*	1.38	1.76	0.336	0.679
*Selenomonas*	2.14	1.13	0.001	0.071
*Sharpea*	2.14	1.50	0.336	0.679
*Sporobacter*	2.37	1.61	0.121	0.516
*Streptococcus*	6.90	5.29	0.094	0.479
*Subdivision5 genera incertae sedis*	2.21	2.10	0.536	0.797
*Succiniclasticum*	2.93	3.06	0.694	0.899
*Succinivibrio*	0.35	1.70	0.000	0.053
*Syntrophococcus*	0.91	0.56	0.014	0.251
*Treponema*	4.48	7.07	0.054	0.402

*BP35 = Concentrate formulated with 35% by-product. BP95 = Concentrate formulated with 95% by-product. *P* values were generated using the Wilcoxon rank sum test, with correction for FDR using the BH method. Adjusted (adj) *P* values deemed significant when (*P* < 0.05).*

BP inclusion rate affected the abundance of *Selenomonas* (adj *P* = 0.071) and *Clostridium IV* (adj *P* = 0.053) with both genera tending to have a greater abundance in the BP35 dietary treatment (Table 4). Although numeric differences were apparent in the average relative abundances, BP inclusion rate had no significant influence on the abundance of methanogens when calculated relative to all members of the microbial communities.

#### Members of the Firmicutes and Proteobacteria Phyla Associated With Diet Digestibility and VFA Production

The abundance of the *Firmicutes* phylum had a strong positive correlation with the total DM digestibility (DMD) (r_s_, 0.84; *P* < 0.001), CP digestibility (CPD) (r_s_, 0.83; *P* < 0.001), NDF digestibility (NDFD) (r_s_, 0.86; *P* < 0.0001) and ADF digestibility (ADFD) (r_s_, 0.80; *P* < 0.001). Total (r_s_, 0.63; *P* < 0.02) and all individual VFA production levels had a positive correlation with the abundance of *Firmicutes*.

The relative abundance of *Proteobacteria* had a strong negative correlation with all digestibility parameters [total DMD (r_s_, −0.88; *P* < 0.0001), OMD (r_s_, −0.63; *P* < 0.02), CPD (r_s_, −0.86; *P* < 0.0001), NDFD (r_s_, −0.89; *P* < 0.0001) and ADFD (r_s_, −0.86; *P* < 0.0001)]. A moderate negative relationship was observed for the relative abundance of *Proteobacteria* with total VFA (r_s_, −0.60; *P* < 0.02) and propionate (r_s_, −0.58; *P* < 0.03) production. A strong negative relationship with butyrate (r_s_, −0.76; *P* < 0.01) and a near significant association with acetate (r_s_, −0.51; *P* = 0.052) production, was also observed. The relative abundance of *Succinivibrionaceae* had a negative correlation with all digestibility parameters. Total VFA production had a significant negative association (r_s_, −0.52; *P* < 0.05) with *Succinivibrionaceae* with both propionate (r_s_, −0.53; *P* < 0.05) and butyrate (r_s_, −0.68; *P* < 0.01) production observed to a have a moderately negative association.

### Relationship Between Members of the Microbial Community With Daily Methane Production

The associations between members of the microbial community and methane production were investigated in an effort to better understand the microbial factors resulting in the divergence of methane production. At lower taxonomic levels methanogen abundance was found to be associated with methane production. For example a strong positive relationship between *Methanobrevibacter* and daily methane output (r_s_, 0.63; *P* < 0.02) was observed. In addition, there was a tendency for a similar correlation between *Methanobacterium* and methane output (r_s_, 0.46; *P* = 0.081).

Within the bacterial proportion, the abundance of *Proteobacteria* was found to be negatively associated with methane production when expressed on a daily basis (r_s_, −0.71; *P* < 0.01) or per unit of OMD (r_s_, −0.65; *P* < 0.01). At lower taxonomic levels, the relationship of members of the *Proteobacteria* phylum with methane production was maintained for the mean abundance of *Succinivibrio* (r_s_, −0.68; *P* < 0.01). The abundance of the *Firmicutes* phyla was found to be positively correlated with both daily methane production (r_s_, 0.79; *P* < 0.001) and output per unit of OMD (r_s_, 0.75; *P* < 0.01). Similarly this relationship was maintained for the genera *Selenomonas* (r_s_, 0.82; *P* < 0.001) and *Clostridium IV* (r_s_, 0.73; *P* < 0.01) with daily methane output.

## Discussion

Findings from this *in vitro* study have shown a 22.7% reduction in methane output with the supplementation of a concentrate formulated exclusively from DDGS, PKE and SHs to a pasture based diet, in comparison to a barley and soybean meal based concentrate formulation. The observed differences in all digestibility parameters associated with the BP95 diet, most likely originated from the varied supply of fiber (NDF), starch, and fat between the diets (Table 1). Indeed, fiber has a lower digestibility in comparison to starch (Getachew et al., 2004) with some components of our BP mixture previously shown to be moderately degradable (Ipharraguerre and Clark, 2003; Schingoethe et al., 2009; Wang et al., 2018). In addition, fat supplementation is known to reduce fiber degradation in the rumen (Patra, 2013) further explaining the reduced digestibility observed in the high fiber BP95 diet. Furthermore, the limited fermentation of lipids by ruminal microbes (Nagaraja et al., 1997) likely contributed to the reduced diet digestibility, VFA and daily methane production in the BP95 diet, as fat replaced fermentable OM. However, the proportions of individual VFAs were similar across both diets, with the exception of butyric acid.

Findings from this study are in agreement with the proposed relationship between the tandem reduction in methane production and H recovery (Ungerfeld, 2015). However, some 45% of H was unaccounted for in our analysis, which may have been redirected to other H sinks not investigated in this study, such as H_2_ and additional fermentation end products beyond the predominant VFAs and methane, as proposed by previous authors (Machmüller et al., 1998; Dohme et al., 2000; Guyader et al., 2017).

While differences in fat, starch and NDF content between the two diets is accepted to have influenced daily methane production, it is more precise to estimate methane output, *in vitro*, per unit of substrate digested (Yáñez-Ruiz et al., 2016). After correcting methane output for OMD, dietary ranking was consistent with a 20.8% reduction in output detected in the BP95 diet. As methane is a product of microbial fermentation (Tapio et al., 2017) differences in the components of the diets likely altered the composition of the microbial communities, which may have contributed to the observed variation in methane output per OMD between the diets.

An increase in the proportion of dietary unsaturated fat has been shown to increase the abundance of *Succinivibrionacea* and *Succinivibrio* in cattle and sheep (Huws et al., 2015; Lyons et al., 2017). Therefore the increased abundance of some members of the *Succinivibrionacea* family in the BP95 diet may be due to the elevated supply of lipids, some of which may have been unsaturated and originated from DDGS (Castillo-Lopez et al., 2014; Xu et al., 2014). In addition, the non-structural carbohydrate component of SHs is high in pectin (Makkar, 2012) which may have benefitted some members of the *Succinivibrionacea* family capable of degrading pectin (Stewart et al., 1997).

Bacteria belonging to the phylum, *Firmicutes*, such as members of the *Lachnospiraceae* and *Ruminococcaceae* family are known degraders of cellulose and hemicellulose (Krause et al., 2003; Dai et al., 2015; Huws et al., 2016; Mayorga et al., 2016) while others are accepted producers of CO_2_ and H_2_ (Marounek and Dušková, 1999; Rooke et al., 2014). In agreement with the literature, the abundance of *Firmicutes* was strongly correlated with NDF digestibility and daily gas production. In addition, it is likely the strong relationship between the abundance of *Firmicutes* and methane output (daily and per unit of OMD) was as a result of the fibrolytic capabilities of members of the phyla.

The observed reduction in the Firmicutes:Bacteroidetes ratio in the BP95 is in agreement with the effects of DDGS dietary inclusion, as presented by others (Callaway et al., 2010; Ramirez et al., 2012). Equally, the *Firmicutes* phylum is likely to have been impacted by the increased fat content in the BP95 diet, with some members of the phylum found to be adversely impacted by lipids (Maia et al., 2007). SHs have also been shown to reduce the abundance of *Firmicutes in vitro* (Wang et al., 2018). As a result, dietary related effects to the abundance of the *Firmicutes* phyla most likely resulted in a varied supply of CO_2_ and H_2_ to hydrogenotrophic methanogens. Finally, the abundance of *Selenomonas* likely benefited from the higher starch content observed in the BP35 with previous studies showing an elevated abundance of the bacteria in high grain diets (Fernando et al., 2010).

In this study, a high relative abundance of archaea was observed across both dietary groups at all taxonomic levels, in comparison to other RUSITEC and *in vivo* experiments, utilizing the same primers to simultaneously target bacterial and archaeal populations (Duarte et al., 2017; Bowen et al., 2020). However, archaea have previously been reported as having a relative abundance of greater than 7% in feed restricted animals (McCabe et al., 2015) and thus similar to the proportion observed in this study. In addition, comparisons between other RUSITEC experiments are difficult due to the separate reporting of archaeal and bacterial relative abundances by some groups (Belanche et al., 2016a, b). Furthermore, sampling time may have had an effect on the abundance of methanogens, with both the abundance of bacteria and archaea shown to peak 2–4 h after the introduction of feed to the RUSITEC (Belanche et al., 2013). As samples were collected 24 h after feeding, potentially a greater post feeding decrease in bacteria, relative to the archaea, may have occurred resulting in the high relative abundance of archaea observed in our study.

Both PKE and DDGS have previously been shown to negatively affect the methanogen population in goats (Abubakr et al., 2014) and cattle (Zhou et al., 2012). However, in this study the relative abundance of the methanogen proportion of the microbial community was not affected by diet. *McrA* gene expression has previously been shown to be associated with methane emissions (Shi et al., 2014) and is noted as a more credible methanogenesis biomarker than 16S rRNA based methods (Wilkins et al., 2015). Therefore the effect of diet on abundance of *Firmicutes* may have resulted in a reduced supply of H_2_ and CO_2_, and subsequently influenced the expression of methanogenesis pathways resulting in the reduced methane production observed in the BP95 diet.

Findings from this *in vitro* study highlight the methane abatement potential of a low starch but high fat and fiber formulated concentrate, for grass based ruminant production systems. Data generated from our metataxonomic approach, suggests the rumen prokaryotic composition to be altered in favor of a potential reduction in the production of methanogenesis substrates, with the supplementation of our 95% BP formulated concentrate. However, confirmation *in vivo* will be required to confirm the methane abatement potential, of concentrates formulated solely from SHs, PKE and DDGS to act a cost effective dietary mitigation strategy for pasture based livestock production.

## Data Availability Statement

The datasets presented in this study can be found in online repositories. The names of the repository/repositories and accession number(s) can be found at: https://www.ncbi.nlm.nih.gov/, PRJNA610813.

## Ethics Statement

The animal study was reviewed and approved by The animal research ethics committee (AREC) at University College Dublin (UCD).

## Author Contributions

AK, TB, and JH conceived and designed the experiments. PS and JH performed the experiments. PS, JH, and AK analyzed the data. AK, JH, SW, DK, and TB contributed reagents, materials, and analysis tools. PS, AK, SW, DK, TB, and JH interpreted results and wrote the manuscript. All authors contributed to the article and approved the submitted version.

## Conflict of Interest

The authors declare that the research was conducted in the absence of any commercial or financial relationships that could be construed as a potential conflict of interest.

## Footnotes

1 https://benjjneb.github.io/dada2/training.html
